# Cortical signatures in behaviorally clustered autistic traits subgroups: a population-based study

**DOI:** 10.1038/s41398-020-00894-3

**Published:** 2020-06-27

**Authors:** Angeline Mihailov, Cathy Philippe, Arnaud Gloaguen, Antoine Grigis, Charles Laidi, Camille Piguet, Josselin Houenou, Vincent Frouin

**Affiliations:** 1 Neurospin, Institut Joliot, CEA, Université Paris-Saclay, Gif-sur-Yvette, 91191 France; 2 CNRS-Centrale Supélec, 3 rue Joliot-Curie, 91192 Gif-sur-Yvette, France; 3 APHP, Mondor Univ. Hospitals, DMU IMPACT, INSERM, U955, Translational Neuropsychiatry Team, University of Paris-Est Créteil, 94000 Créteil, France; 4 Faculty of Medicine, University of Geneva, Geneva, Switzerland

**Keywords:** Diagnostic markers, Human behaviour, Neuroscience, Prognostic markers, Autism spectrum disorders

## Abstract

Extensive heterogeneity in autism spectrum disorder (ASD) has hindered the characterization of consistent biomarkers, which has led to widespread negative results. Isolating homogenized subtypes could provide insight into underlying biological mechanisms and an overall better understanding of ASD. A total of 1093 participants from the population-based “Healthy Brain Network” cohort (Child Mind Institute in the New York City area, USA) were selected based on score availability in behaviors relevant to ASD, aged 6–18 and IQ >= 70. All participants underwent an unsupervised clustering analysis on behavioral dimensions to reveal subgroups with ASD traits, identified by the presence of social deficits. Analysis revealed three socially impaired ASD traits subgroups: (1) high in emotionally dysfunctional traits, (2) high in ADHD-like traits, and (3) high in anxiety and depressive symptoms. 527 subjects had good quality structural MRI T1 data. Site effects on cortical features were adjusted using the ComBat method. Neuroimaging analyses compared cortical thickness, gyrification, and surface area, and were controlled for age, gender, and IQ, and corrected for multiple comparisons. Structural neuroimaging analyses contrasting one combined heterogeneous ASD traits group against controls did not yield any significant differences. Unique cortical signatures, however, were observed within each of the three individual ASD traits subgroups versus controls. These observations provide evidence of ASD traits subtypes, and confirm the necessity of applying dimensional approaches to extract meaningful differences, thus reducing heterogeneity and paving the way to better understanding ASD traits.

## Introduction

Autism spectrum disorder (ASD) is a complex array of neurodevelopmental conditions typically characterized by social interaction and communication impairments, and restricted and repetitive behaviors^1^. The heterogeneity of ASD, reflected in its etiology, development, and biological phenotypes, presents an enormous challenge in the delineation and understanding of the disorder. It is therefore fundamental to define distinct subgroups of ASD, and dimensional approaches have been proposed as one way to perform this.

It has been suggested that autistic traits, in particular social and communication deficits, are distributed along a continuum extending into the general population^2–5^. Core autistic traits are often concomitant with behavioral traits varying in type and degree, thus further complicating the characterization of ASD. Common symptoms reported in ASD patients include anxiety^6^, depressive symptoms^7^, aggression^8^, attention deficits^9^, hyperactivity^10^, and sleep difficulties^11^. ASD patients are also more likely to present medical issues including seizures^12^, immune system abnormalities^13^, and gastrointestinal disorders^14^. Behavioral symptoms often indicate the presence of comorbid psychiatric disorders such as attention-deficit/hyperactivity disorder (ADHD), major depressive disorder, anxiety disorders, and conduct disorders. This complexity enforces the legitimacy of implementing a dimensional approach to examine continuous autistic traits and fluctuating behavioral symptoms as inter-correlated constructs varying in expression. A dimensional approach can promote stratification according to behavioral and biological features as suggested by the Research Domain Criteria^15^.

A prominently studied biological feature in ASD is cortical morphometry. In particular, ASD is accompanied by a range of aberrant cortical patterns present in both volumetric and surface-based morphology studies^16,17^. Several case-control studies have reported various changes in thickness including increases^16,18^ and decreases^19,20^. Surface area in ASD has been less investigated, with most results reporting no differences^16,21,22^. Studies have also reported significant global increases in surface area at younger ages^23,24^, as well as decreases in later stages of life^24^. Gyrification observations within ASD on the other hand have greatly reported increases^17,21,25,26^, with few showing decreases^27,28^. Though neuroanatomical investigations in ASD have remained largely unreplicated, few observations have persisted. This includes early brain overgrowth in frontal and temporal lobes^29–31^, causing children to achieve a nearly developed brain volume earlier than controls. Also, longitudinal studies in cortical thickness have shown a general trajectory of accelerated thinning with age in ASD patients versus controls in frontal, temporal, and parietal areas^22,32^. We presume that the general variability and lack of reproducibility is due to the frequently encountered practice of combining heterogeneous ASD patients into one group within case-control studies.

The objective of the present study is to thus disentangle and better understand the behavioral heterogeneity in ASD by using subjects with autistic traits to extract refined cortical morphometry features. To observe how behavioral dimensions distribute in a general population of children and adolescents that vary in degree of social impairment spreading across the spectrum, we designed the following experiment. We chose a broad age range (5–18), including males and females, within a large-scale multidimensional population-based cohort in order to capture a larger effect variance (compared to a purely ASD cohort) by focusing on behavioral constructs, and not an ASD diagnosis. We then conducted an unsupervised clustering analysis on the z-scores of several behaviors, especially chosen due to their manifestation in ASD, in order to isolate data-driven subgroups high in our dimension of interest: social impairment, which is a surrogate of autistic traits^3,33^. Extracted subgroups were subsequently combined into one autistic traits group and compared in cortical surface features (thickness, gyrification, and surface area) to the remainder of the population (i.e., the remaining subgroups that do not exhibit high social impairments, serving as our controls), thus mimicking a case-control study. We show that the case-control paradigm does not extract meaningful cortical features and that behavioral stratification is required. Therefore, in order to achieve clinically relevant morphometric signatures, we ran morphological analyses comparing each of our isolated socially impaired subgroups to controls. This should provide us with a better understanding of underlying heterogeneity present in the physiology of autistic traits, and ultimately ASD.

## Materials and methods

### Part 1: Clinical profiles

#### HBN cohort and participants

The Healthy Brain Network (HBN) cohort initiative within the Child Mind Institute began in 2015 with the goal of collecting brain imaging, cognitive/behavioral, and genetic data for 10,000 children and adolescents (5–21 years old) to investigate the heterogeneity behind neuropsychiatric and neurocognitive development^34^. It comprises a population of individuals at-risk for developing psychiatric disorders and typically developing participants. Subjects were recruited through flyer dissemination and subsequently assessed on clinical questionnaires at three sites in New York City, USA: Staten Island, Mobile Van, Midtown.

In the status of the HBN cohort, consensus diagnostics are not available for most of the subjects enrolled; however, this does not preclude the possibility to carry out our dimensional study since subjects were not selected based on an ASD diagnosis, but rather on the presence of behavioral constructs relevant in the field of ASD. There were 1800 subjects participating at the time of this study, of which 1093 remained based on available overlap in behavioral scores assessing social deficits, hyperactivity, anxiety, irritability, depression, aggression, and attention problems, and having a full-scale Intelligence Quotient (FSIQ) >= 70. We selected these seven behaviors due to their presence in comorbid psychiatric disorders commonly reported in ASD patients, and therefore their frequent emergence along the autistic behavioral spectrum, implicating them in the understanding of ASD behavioral neuropathology^35–39^. Full-scale IQ was measured using the Wechsler Adult Intelligence Scale (WAIS-III, for those over 16) or the Wechsler Intelligence Scale for Children (WISC-III). Written informed consent was obtained from legal guardians and from participants themselves. This cohort study initiative was approved by the Chesapeake Institutional Review Board.

#### Behavioral assessments

One of the most prominent dimensions in ASD patients is social impairment. Here, we used data from the widely used 65-item parent social responsiveness scale (SRS) as a quantitative measure of clinical autistic traits, making it the central variable of interest in our study and in fact the score from which we separate out our autistic traits individuals. The SRS has been proven as a valid measure of autistic traits and thus has been used as a measure of autistic traits (for the purpose of understanding ASD) in several behavioral, genetic, and neuroimaging studies^3,28,32,33,40–44^. Though not a diagnostic tool, the SRS exhibits high inter-rater and cross-cultural reliability, and correlates greatly with the Autism Diagnostic Observation Schedule (ADOS) and the Autism Diagnostic Interview—Revised (ADI-R) diagnostic assessments for ASD from the DSM-5, making it a robust measure to use in the dimensional study of ASD behaviors^3,33,40,45^. Similarly, for the remaining behaviors we did not use diagnostic assessments but rather scales measuring behavioral trait severity. Hyperactivity levels were determined using the hyperactivity subscale within the Strengths and Difficulties Questionnaire (SDQ)^46^; anxiety was measured using the total score from the Screen for Child Anxiety Related Disorders Parent-Report (SCARED-P)^47^; irritability was defined using the total score of the Affective Reactivity Index Parent-Report (ARI-P)^48^; and lastly, levels of depression, aggression, and attention problems were determined using subscales of the same names within the Child Behavioral Checklist (CBCL)^49^.

#### Unsupervised clustering analysis (K-means)

We conducted a k-means analysis on scaled z-scores of the previously mentioned 7 behaviors. This extracted subgroups varying in SRS and other accompanying behavioral characteristics. Briefly, k-means is an algorithm identifying mean cluster centroids, which serves to partition a sample into k subgroups^50^. A substantial challenge in such analyses lies in determining the number of clusters, which is a user-defined parameter. To address this problem, the chosen number of clusters k was determined using a Bayesian Information Criterion (BIC) distribution (Supplementary Fig. 1)^51^.

Mean behavioral scores, FSIQ and age were compared between subgroups using non-parametric 2-sided Mann–Whitney *U* tests, while gender differences were determined using a chi-square test. Python version 2.7 and R 3.4.0 were used on a Linux platform to perform all analyses in this study. Python packages used include Pandas (version 0.19.2), SciPy (version 1.1.0), and Matplotlib (version 1.5.1).

### Part 2: Neuroimaging analysis of cortical surface features

#### Structural MRI acquisition and processing

MRI acquisition took place at three different sites: mobile 1.5T Siemens Avanto in Staten Island, 3T Siemens Tim Trio at Rutgers University Brain Imaging Center, and 3T Siemens Prisma at the CitiGroup Cornell Brain Imaging Center (acquisition protocols are extensively described in Alexander et al. ^34^).

T1-weighted images were processed using the FreeSurfer software version 6.0.0 (https://surfer.nmr.mgh.harvard.edu/). For more information on precise methods of image analysis and the construction of anatomical information for each individual done by this software, refer to^52,53^. Briefly, the FreeSurfer analysis stream includes intensity normalization, skull stripping, and segmentation of gray (pial) and white matter surfaces^52^. Subsequent tessellation, as well as various topology corrections and inflation, leads to 3D meshes of cortical surfaces in different resolutions. Our work is based on a tessellation with ~160,000 vertices per hemisphere and used the FreeSurfer *fsaverage* template. We focused on three morphological measures of which the processing stream created vertex-wise maps for analysis: cortical thickness (CT), surface area (SA), and gyrification (lGI). The local gyrification index is measured as the ratio between buried and visible cortex^54^. All images were manually inspected in-house, in addition to using the Euler number as a metric of quality by retaining images at a threshold of -217, as specified in Rosen et al. ^55^.

#### Elimination of site effects on cortical features using ComBat

A harmonization process was performed to account for the multiple acquisition sites. Features extracted from structural MR images are prone to technical variability across acquisition centers such as differences in scanning parameters, scanner manufacturers and field strengths. In order to remove cortical feature bias and variability caused by the unwanted site effects, the ComBat technique was applied to harmonize feature data along our three acquisition centers. This method adjusts the mean value and variance of feature measures across sites^56^.

#### Statistical analysis

Vertex-wise statistical analyses were conducted using the command-line group analysis stream in FreeSurfer. Cortical surfaces for each participant were first registered to a study-specific template, then smoothed using a full-width-at-half maximum (FWHM) kernel of 10 mm for CT and SA, and 5 for lGI. A general linear model was fit at each vertex *i* to compare the three morphological measures between groups, using gender as a categorical covariate, and age and FSIQ as continuous covariates (site effects were already accounted for at the vertex level), and including the residual error:

$${\it{y}}_i = \beta _{\mathrm{0}} + \beta _{\mathrm{1}}{\mathrm{Group}} + \beta _{\mathrm{2}}{\mathrm{Sex}} + \beta _{\mathrm{3}}{\mathrm{Age}} + \beta _{\mathrm{4}}{\mathrm{FSIQ + }}\varepsilon _{\mathrm{i}}.$$

We performed a cluster-level analysis using a cluster-forming threshold of *p* = 0.01. We report clusters with cluster-wise *p*-value (cwp) of cwp <0.05. These *p*-values were corrected for multiple comparisons using the *mri_glmfit-sim* precomputed MonteCarlo simulation.

## Results

### Part 1: Clinical profiles

#### Data-driven behavioral subgroups in HBN cohort

**Fig. 1:**
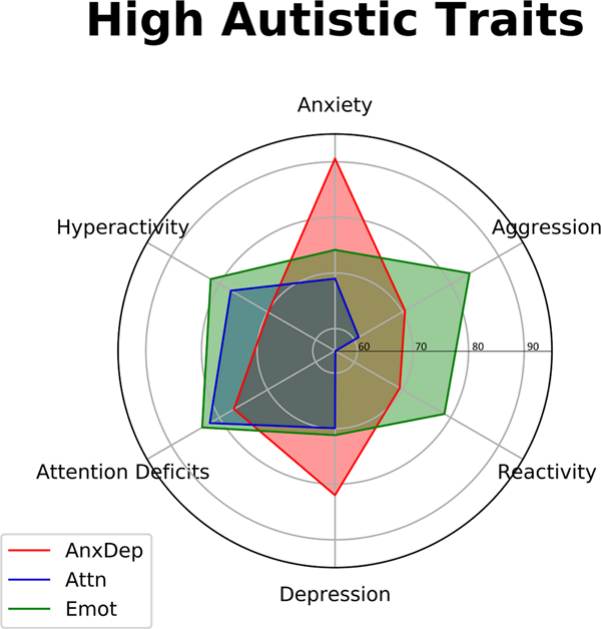
Radar plot of high autistic traits subgroups. A clustering analysis yielded nine subgroups varying in behavioral composition. From these, three exhibited high SRS levels. The first subgroup (*Emot*), colored in green, had strong emotional dysregulation (aggression and reactivity) with ADHD-like symptoms. The second subgroup (Attn), colored in blue, showed ADHD-like tendencies. Lastly, the third subgroup (AnxDep), colored in red, exhibited high levels of anxiety and depression, as well as attention deficits. This plot was built upon normalized scores that were converted to a scale of 1 to 100 (as indicated by each encircling gray line) for simplification.

Based on the computed BIC value distribution, a *k* value of 9 was retained as our supervised partitioning for this study (Supplementary Fig. 1). Thus, upon running the clustering analysis, we obtained nine subgroups with various behavioral profiles (Table 1). The average SRS levels were used to decide which subgroups represented high autistic traits participants and which were controls. From these nine subgroups, three expressed high levels of SRS, representing our socially impaired “high autistic traits” subgroups. The SRS levels of these three subgroups fall within the “*severe*” or at least upper “*moderate*” classification of the SRS scale (an SRS value above ~80), thus indicating a high level of social impairment, providing us with greater confidence that subjects within these subgroups have “autistic-like” traits (Supplementary Fig. 2). Additionally, mean SRS values in these three subgroups are comparable to the average SRS level of ~86 reported in diagnosed ASD patients^33,57,58^. Regarding the behavioral compositions of our three high autistic traits subgroups, one subgroup showed high levels of reactivity, aggression, and ADHD-like symptoms (hyperactivity and attention issues), *n* = 107 (described as emotional dysregulation—*Emot*); the second maintained normal levels in all behavioral scores except for attention problems and hyperactivity, *n* = 82 (described as attention problems—*Attn*); and the third showed high levels of anxiety and depression, as well as attention deficits, *n* = 61 (described as anxiety depression—*AnxDep*) (Fig. 1). Clinically high levels were determined for each behavioral measure according to the literature^3,47,48,59–61^. Though the remaining six subgroups contained subjects with SRS values ranging from low to high, each of these subgroups maintained an overall low SRS mean and were thus combined as our control group (*n* = 843) with the aim of creating a representative general population without autistic traits subjects. Studies often barely obtain additional behavioral information on their controls other than a “non-diagnosis” or “low SRS”. By combining these remaining six subgroups into one control group, we smooth out several behavioral heterogeneities and yield a control group composed of a wide behavioral spectrum, while still maintaining low mean levels of SRS (our target variable of interest to be contrasted in subsequent analyses) (Supplementary Fig. 3).

**Table 1:** Nine resultant subgroups from clustering analysis.

Subgroups	*n* (Total = 1093)	SRS (s.d.): Social Deficits	SCARED-p (s.d.): Anxiety	ARI-p (s.d.): Reactivity	SDQ-Hyperactivity (s.d.)	CBCL-AB (s.d.): Aggression	CBCL-AP (s.d.): Attention Problems	CBCL-WD (s.d.): Depression	Mean Age (s.d.)	Gender Ratio	Mean FSIQ (s.d.)
**Subgroup 1 (AnxDep)**	61	88.7* (26.0)	40.0* (13.0)	6.1 (3.0)	5.9 (1.9)	12.6 (3.8)	10.8* (**3.4)**	8.4* (**3.1)**	12.7 (2.7)	35:26	96.9 (14.2)
Subgroup 2	221	24.4 (13.8)	6.7 (5.1)	0.8 (1.4)	2.0 (1.5)	1.9 (2.3)	2.1 (2.1)	0.8 (1.2)	11.2 (2.9)	114:107	101.9 (15.2)
Subgroup 3	124	45.6 (16.2)	22.8 (10.2)	2.8 (2.1)	3.1 (1.7)	5.4 (3.6)	4.9 (3.0)	4.5 (2.5)	12.3 (3.1)	59:65	100.1 (15.6)
**Subgroup 4** (**Attn)**	82	93.5* (**22.5)**	20.0 (9.4)	2.2 (1.9)	7.5* (**1.9)**	7.4 (4.0)	12.6* (**2.7)**	5.5 (2.4)	11.7 (3.1)	57:25	93.3 (15.3)
Subgroup 5	130	53.3 (18.3)	11.6 (7.7)	7.5 (1.9)	6.0 (1.9)	14.0 (5.0)	8.7 (2.8)	2.6 (2.0)	10.6 (2.9)	90:40	99.5 (14.5)
Subgroup 6	141	30.6 (11.5)	5.9 (5.0)	1.1 (1.5)	6.2 (1.6)	3.9 (3.1)	8.0 (2.6)	0.9 (1.0)	10.8 (2.9)	99:42	98.7 (15.1)
**Subgroup 7 (Emot)**	107	98.3* (**20.7)**	24.7 (11.8)	8.7* (**2.3)**	8.4* (**1.5)**	20.1* (**5.0)**	13.3* (**3.4)**	5.9 (3.0)	10.7 (2.8)	71:36	95.7 (15.4)
Subgroup 8	104	55.5 (17.9)	14.3 (9.6)	3.0 (2.0)	8.6* (**1.4)**	10.1 (4.6)	12.9* (**2.3)**	1.7 (1.5)	10.1 (2.3)	78:26	98.3 (15.0)
Subgroup 9	123	63.3 (16.9)	14.0 (7.4)	1.7 (1.7)	5.9 (1.4)	4.4 (2.9)	6.9 (2.1)	2.0 (1.6)	10.6 (2.8)	77:46	96.1 (15.6)

*Values surpassing high clinical levels of each score.

Ranges for each score: SRS: 0 to 123+; SCARED-p: 0 to 82; ARI-p: 0 to 12; SDQ-Hyperactivity: 0 to 10; CBCL-AB: 0 to 40+; CBCL-AP: 0 to 22+; CBCL-WD: 0 to 17+. Age ranges: Subgroup 1 (AnxDep): 6.9 to 17.1; Subgroup 2: 6.0 to 17.7; Subgroup 3: 5.8 to 17.9; Subgroup 4 (Attn): 6.6 to 17.7; Subgroup 5: 5.8 to 17.6; Subgroup 6: 6.0 to 17.7; Subgroup 7 (Emot): 6.1 to 17.3; Subgroup 8: 6.2 to 16.4; Subgroup 9: 6.1 to 17.6.

Mean behavioral scores and demographic data for each subgroup, including our three high autistic traits subgroups of interest (highlighted). Behavioral scores with a clinically high threshold were bolded and denoted with a “*” superscript (s.d = Standard Deviation).

Upon comparing the three socially impaired subgroups to one another, several significant differences in behavioral scores were found (Table 2a, “*Comparisons between subgroups*”). We also compared each autistic traits subgroup to controls and observed significant differences in all behavioral scores, except for reactivity in the *Attn* subgroup. Lastly, we decided to combine all three high autistic traits subgroups into one “autistic-like” group (combined high SRS, *hSRS*) to compare against controls, which yielded highly significant differences in every behavioral score (Table 2a, “*Comparisons to controls*”*)*.

**Table 2:** Between-group behavioral score and demographic comparisons.

	SRS: Social Deficits	SCARED-p: Anxiety	ARI-p: Reactivity	SDQ-Hyperactivity	CBCL-AB: Aggression	CBCL-AP: Attention Problems	CBCL-WD: Depression	Age	Gender (*X*^2^)	FSIQ
**A. Behavioral data cohort (*n*=1093)**										
**Comparisons between subgroups**										
Emot vs. Attn	0.07	**4.21** × 10−3*	**7.30** × 10−30*	**5.43** × 10−4*	**1.85** × 10−29*	0.053	0.15	0.014*	0.32	0.13
Emot vs. AnxDep	0.01*	**1.77** × 10−11*	**3.52** × 10−8 *	**4.58** × 10−14*	**3.80** × 10−17*	**2.03** × 10−5*	**1.43** × 10−6*	**6.80** × 10−6*	0.76	0.25
Attn vs. AnxDep	0.16	**2.72** × 10−16*	**4.72** × 10−14*	**1.41** × 10−6*	**8.13** × 10−12*	**1.70** × 10−3*	**5.92** × 10−9*	0.02*	0.19	0.056
**Comparisons to controls**										
Emot	**3.71** × 10−56*	**1.68** × 10−26*	**3.33** × 10−50*	**8.07** × 10−34*	**1.26** × 10−54*	**1.19** × 10−39*	**3.17** × 10−36*	0.15	0.37	**8.94** × 10−3*
Attn	**3.75** × 10−41*	**3.57** × 10−14*	0.37	**3.48** × 10−17*	**1.15** × 10−4*	**2.49** × 10−30*	**5.38** × 10−31*	0.03*	0.18	**2.01** × 10−4*
AnxDep	**4.92** × 10−27*	**8.92** × 10−34*	**8.47** × 10−17*	**7.51** × 10−4*	**7.58** × 10−19*	**9.30** × 10−14*	**2.68** × 10−34*	**2.69** × 10−6*	0.64	0.13
hSRS (Emot + Attn + AnxDep)	**1.68** × 10−102*	**1.19** × 10−56*	**2.44** × 10−38 *	**5.40** × 10−40*	**1.91** × 10−53*	**2.15** x 10−67*	**4.18** x 10−81*	**7.14** x 10−3*	0.3	**7.06** x 10−5*
**B. Neuroimaging cohort (*n*** = **527; subset of behavioral data cohort)**										
**Comparisons between subgroups**										
Emot vs. Attn	0.09	0.49	**1.81** × 10−14*	**4.01** × 10−3*	**5.95** × 10−15*	0.35	0.24	**4.49** × 10−3*	0.76	0.21
Emot vs. AnxDep	0.02*	**3.27** × 10−7*	**2.28** × 10−5*	**2.96** × 10−7*	**1.08** × 10−10*	**4.61** × 10−2*	**3.93** × 10−4*	**4.36** × 10−4*	0.99	0.29
Attn vs. AnxDep	0.12	**1.88** × 10−7*	**2.38** × 10−7*	**4.14** × 10−3*	**8.12** × 10−8*	0.08	**5.83** × 10−4*	0.06	0.66	0.11
**Comparisons to controls**										
Emot	**2.41** × 10−26*	**3.03** × 10−9*	**1.93** × 10−23*	**8.93** × 10−17*	**2.05** × 10−26*	**3.19** × 10−18*	**1.23** × 10−15*	0.09	0.31	0.04*
Attn	**9.22** × 10−20*	**1.26** × 10−8*	0.31	**8.18** × 10−9*	**1.15** × 10−3*	**2.19** × 10−15*	**8.80** × 10−17*	**4.93** × 10−3*	0.12	**1.86** × 10−3*
AnxDep	**4.32** × 10−13*	**5.01** × 10−17*	**1.84** × 10−9*	**2.35** × 10−3*	**3.01** × 10−12*	**1.94** × 10−9*	**2.14** × 10−17*	**5.34** × 10−5*	0.61	0.16
hSRS (Emot + Attn + AnxDep)	**2.33** × 10−47*	**3.10** × 10−25*	**4.53** × 10−19*	**1.75** × 10−20*	**6.96** × 10−29*	**1.09** × 10−33*	**1.15** × 10−38*	**8.25** × 10−3*	0.07	**1.15** × 10−3*

*Significant *p*-values.

Demographic and behavioral score comparisons, presented as p-values, between autistic traits subgroups, and between each autistic traits subgroup and controls. The top section shows these comparisons in the behavioral cohort *(*“*A. Behavorial data cohort (n* = *1093)*”, with autistic traits subgroups (i.e. *Emot, Attn, AnxDep*) compared to each other (under “*Comparisons between subgroups*”), each autistic traits subgroup compared to controls, and finally a combination of all three autistic traits subgroups (hSRS) compared to controls (under “*Comparisons to controls*”). The bottom section (*B. Neuroimaging cohort (n* = *527; subset of behavioral data cohort))* presents the same comparisons in the neuroimaging cohort, which is composed of a subset of subjects from the behavioral cohort with usable structural MRI data. Significant *p*-values are bolded with a “*” superscript.

With respect to demographic information, there were no significant differences in gender and FSIQ between all subgroups. However, there were reported differences in age, though age ranges were similar (mean age = 10.8, SD = 3.4) (Table 2a, “*Comparisons between subgroups*”). Upon comparing each subgroup to controls, we again found no differences in gender. We did however find differences in age between all subgroups and controls, except for *Emot*. Although age differences were present, age ranges were again similar. FSIQ differed between all subgroups and controls, except for *AnxDep*, which is to be expected since autistic traits are generally accompanied by differences in FSIQ. Lastly, the comparison between the *hSRS* group and controls yielded significant differences in age and FSIQ, but not gender (Table 2a, “*Comparisons to controls*”*)*. Due to these differences, we deemed it important to control for FSIQ, age and gender in the subsequent neuroimaging analysis.

### Part 2: Neuroimaging analysis of cortical surface features

#### Morphological comparisons

**Fig. 2:**
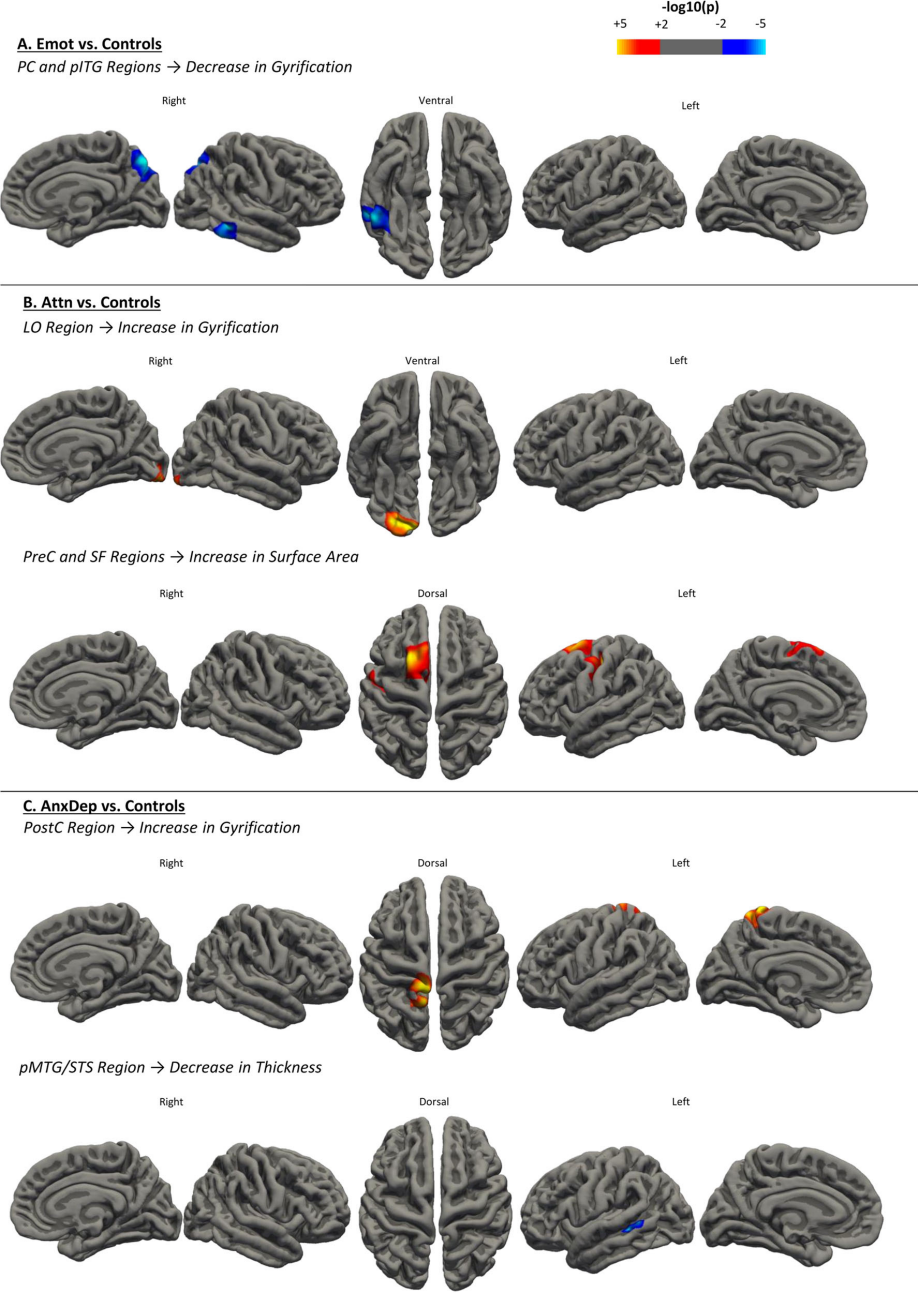
Surface feature comparisons between each subgroup and controls. **a** The *Emot* subgroup yielded decreases in gyrification in the right precuneus and temporal regions (cwp = 0.0004 and 0.005, respectively). **b** The *Attn* subgroup exhibited increases in gyrification in the left lateraloccipital region (cwp = 0.002), and increases in surface area in the left precentral and superiorfrontal regions (cwp = 0.02 and 0.02, respectively). **c** The *AnxDep* subgroup showed increases in gyrification in the left postcentral area (cwp = 0.02), and decreases in thickness in the left middletemporal gyrus/superior temporal sulcus (cwp = 0.04). Colors represent the –log_10_(*p*-value), with red(+) indicating an increase and blue(−) indicating a decrease compared to controls in affected morphological features.

After removing subjects that have not undergone MRI acquisition and/or did not pass the T1 image quality check, as well as those removed during the outlier detection step (Supplementary Fig. 4), we obtained a sample of: *n* = 47 in the “*Emot*” group, *n* = 39 in the “*Attn*” group, *n* = 31 in the “*AnxDep*” group, and *n* = 410 controls (Supplementary Table 1), producing a total of 527 subjects with available T1 data participating in the study. Behavioral score and demographic information comparisons were nearly identical to the behavioral cohort (Table 2b, “*Neuroimaging cohort’*”). To delineate the interest and significance of subtyping in an autistic traits population, we first combined all three subgroups into one large group (*hSRS*, *n* = 117) and compared cortical thickness, local gyrification and surface area against controls. Indeed, this comparison did not yield significant differences in any of the measured surface features. We then compared the same surface features between each of our three subgroups against controls. After correction for multiple comparisons, the *Emot* subgroup exhibited decreases in gyrification in the right hemisphere in two separate clusters, one spanning the precuneus (including parts of the superiorparietal area) (*p* < 0.01, Cohen’s *d* = 0.51), and another in the temporal lobe (including the posterior inferior temporal gyrus and the middletemporal) (*p* < 0.01, Cohen’s *d* = 0.48)(denoted as PC and pITG) (Fig. 2a). The *Attn* subgroup displayed elevated local gyrification peaking in the lateraloccipital area of the right hemisphere (denoted as LO) (*p* < 0.01, Cohen’s *d* = 0.41). Additionally, the *Attn* subgroup also exhibited two separate clusters in the left hemisphere showing increases in surface area in the precentral cortex (along the central sulcus) (*p* < 0.01, Cohen’s *d* = 0.61), and superiorfrontal regions (*p* < 0.01, Cohen’s *d* = 0.58) (denoted as PreC and SF) (Fig. 2b). Lastly, the *AnxDep* subgroup showed increases in gyrification spanning the left postcentral and precuneus regions (PostC) (*p* < 0.01, Cohen’s *d* = 0.33), and decreases in thickness in the left posterior middletemporal gyrus lining the superior temporal sulcus (pMTG/STS) (*p* < 0.01; Cohen’s *d* = 0.55) (Fig. 2c) (Table 3).

**Table 3:** Neuroimaging cluster coordinates information.

Subgroups	Feature	Hemisphere	Cluster name	All areas included in cluster	Peak region	Peak MNI coordinates			Size of region (mm²)	Cluster-wise p-value (cwp)
						*X*	*Y*	*Z*		
**Emot**	↓ Gyrification	Right	**PC** (Precuneus)	Precuneus, Superiorparietal	Precuneus	14	−71.4	40.9	1860.87	**0.0004**
	↓ Gyrification	Right	**pITG** (Posterior Inferior temporal)	Inferiortemporal, Middletemporal	Middletemporal	59	−42.4	−10.4	1237.64	**0.005**
**Attn**	↑ Gyrification	Right	**LO** (Lateraloccipital)	Lateraloccipital, Lingual	Lateraloccipital	26.5	−87.2	−10.7	1395.61	**0.002**
	↑ Surface Area	Left	**PreC** (Precentral)	Precentral, Central Sulcus	Precentral	−32	−18.1	44.2	960.47	**0.02**
	↑ Surface Area	Left	**SF** (Superiorfrontal)	Superiorfrontal	Superiorfrontal	−18.1	10.5	60.8	985.47	**0.02**
**Anxdep**	↑ Gyrification	Left	**PostC** (Postcentral)	Postcentral, Central Sulcus	Postcentral	−8.2	−38.8	74.1	969.46	**0.02**
	↓ Thickness	Left	**pMTG/STS** (posterior Middletemporal/Superior Temporal Sulcus)	Middletemporal, Superior Temporal Sulcus	Middletemporal	−49.1	−39	−5.6	399.23	**0.04**

The type of morphological feature (↑ indicating an increase, and ↓ indicating a decrease), hemisphere, and affected brain regions are indicated above for each autistic traits subgroup. Additional information on MNI coordinate data, region size, peak region and cluster-wise *p*-value for each result within each autistic traits subgroup are also reported.

## Discussion

ASD studies have unceasingly demonstrated heterogeneity, warranting a shift in focus towards initially characterizing these differences before subsequent analysis, and steering away from case-control studies. To this end, a dimensional approach proves most relevant. To the best of our knowledge, this is the first study using an unsupervised clustering analysis on a population-based cohort to investigate how autistic traits cluster with other behavioral dimensions into subgroups, with subsequent isolation of subgroup cortical signatures. Recent evidence advocates that autistic traits fall along a continuum within the general population, which was why this study was not limited to diagnosed individuals, but rather focused on autistic traits as absolute constructs in order to avoid potential selection or environmental biases often accompanying diagnosed patients. We obtained three autistic traits subgroups in our unsupervised clustering analysis with the following behavioral profiles: (1) high aggression, reactivity, and ADHD-like traits (*Emot*), (2) high in attention deficits and hyperactivity (*Attn*), and (3) high in anxiety and depression, as well as attention deficits (*AnxDep*). Furthermore, upon neuroanatomical investigation, we found that comparing each subgroup to controls uncovers unique cortical signatures. Namely, after correcting for multiple comparisons, the *Emot* subgroup showed decreased gyrification in precuneus and posterior inferior temporal regions (PC and pITG); the *Attn* subgroup displayed increases in gyrification in the lateraloccipital (LO) and increases in surface area in the precentral and superiorfrontal regions (SF); and lastly, the *AnxDep* subgroup exhibited an increase in gyrification in the postcentral cortex (PostC), as well as a decrease in thickness in the posterior middletemporal and superior temporal sulcus area (pMTG/STS). Most interestingly, we found that by comparing the structural brain features of one heterogeneous autistic traits group (composed by combing all three subgroups) to controls, we were unable to uncover any cortical signatures. Simply comparing behaviorally diverse ASD-like cases to controls proved far too rudimentary to yield consistent features.

Though several case-control studies have reported neuroanatomical differences in ASD populations, these studies have remained grossly inconsistent, possibly due to ASD heterogeneity. Here, we showed that by running a direct comparison between subjects having high versus low/absent autistic traits, no cortical differences were reported. In a study by Haar et al., authors compared cortical thickness differences between ASD and controls and ran both univariate and multivariate comparisons^62^. Results were strikingly weak and were attributed to the considerable heterogeneity of the ASD population. The authors ultimately suggested that previously reported neuroanatomical differences between cases and controls held low clinical significance, and advocated the necessity of subdividing ASD groups by genetic, clinical and/or behavioral traits in the identification of unique neuroanatomical abnormalities^62^. Further studies in animal research have also encouraged subtyping in ASD, namely a study by Ellegood et al., which ran a clustering analysis on ASD neuroanatomy in a cohort comprising several varieties of mouse models, and subsequently observed resulting clusters’ corresponding gene and behavior patterns^63^. The mentioned studies, along with several others, promote a shift towards subtyping ASD and autistic traits populations in order to better understand and treat the disorder.

Compared to our high vs. low/absent autistic traits contrast that yielded no results, by isolating behaviorally refined autistic traits subgroups we observed cortical signatures despite having lower statistical power than the combined sample. Decreased gyrification detected in the right PC and pITG region in the *Emot* subgroup is consistent with studies in ASD^27,64^. In general, the precuneus is highly implicated in the default mode network (DMN) as well as in visuospatial processing, empathy and memory, while the temporal lobe correlates to memory, audition, theory of mind and visual processes^65–68^. Considering that this group bears high in aggression, studies have also reported a general decrease in gyrification in aggressive patients^69,70^, as well as decreased functional connectivity between the precuneus and other brain regions in patients exhibiting higher aggression traits, possibly due to its role in the DMN and empathy^71,72^. Additionally, this subgroup exhibited high ADHD-like symptoms which have also shown links to precuneus regions of the brain^73,74^. In our second subgroup, *Attn*, we observed increases in surface area in the precentral (primary motor) cortex, which is involved in voluntary motor control^75,76^, and the superiorfrontal gyrus, which is part of the motor control network and also harbors functions in attention, working memory, executive functioning and in the default mode network^74,77,78^. A study has even suggested that early motor impairments are predictors of future social communication delays, further indicating the importance of understanding this region in relation to ASD risk^79^. Specifically, within ASD, atypical motor functioning has been measured in patients from infancy until well into adulthood^80,81^. Seeing as how we observed extensive structural alterations throughout the motor control network in the *Attn* subgroup, this warrants further investigation into the relationship between ADHD-like traits and motor control in ASD and autistic traits populations. The *Attn* subgroup also presented decreases in gyrification in the lateraloccipital region, which is heavily implicated in visual perception, and specifically in face recognition, which greatly influences social communication^25,82,83^. The last subgroup, *AnxDep*, exhibited increases in gyrification in the postcentral region (primary somatosensory cortex), which functions as the main sensory receptive area of the brain^84,85^. In ASD, atypical sensory reception, more specifically over-responsivity to tactile sensory inputs, is a very prevalent symptom^86–88^. This suggests that autistic individuals could easily be overwhelmed, perhaps forging a link to the development of anxious and depressive behaviors, as observed in this subgroup. The *AnxDep* subgroup additionally showed a decrease in thickness in the pMTG/STS region, which has been greatly implicated in language and social aspects, and thus an extremely important cortical region of interest in ASD behavioral studies^89–91^. Therefore, taking into account dimensional constructs of behavior in ASD can better prepare subgroups for the identification of biological mechanisms. Further investigation is warranted into the relationship between affected regions and corresponding subgroup behaviors in the context of ASD since these regions have been consistently reported within the ASD literature.

The behaviors observed in our subgroups enforce the fact that ASD is highly concurrent with several psychiatric conditions in up to 80–95% of patients^35,39^. Reported comorbid disorders include ADHD, depression disorders, anxiety disorders, obsessive compulsive disorder (OCD), and conduct disorders^35–39^. This high degree of comorbidity (based on diagnostic information) corresponds to our dimensional results, which describe these associations in an even more descriptive and spectral manner using behavioral constructs. Having access to a multidimensional cohort containing assessments of behaviors reported in ASD allowed us to explore how core autistic traits inherently distribute with other symptoms in a dimensionally continuous population. By running a data-driven clustering analysis on a population-based cohort, we isolated three main autistic traits subgroups. The *AnxDep* subgroup is composed of subjects high in anxiety, depression and attention deficits. This is in line with findings reported in the literature where anxiety and depression appear to be some of the most common psychiatric comorbidities in ASD patients^38,39^. The *Attn* subgroup could represent an isolated population consisting purely of ADHD and autistic traits in an otherwise behaviorally muted subclass. This may perhaps become the optimal subgroup for studying the overlap between ADHD and ASD. Lastly, the *Emot* subgroup has ADHD-like traits in combination with emotional regulation abnormalities as evidenced by high degrees of aggression and reactivity. The *Emot* subgroup suggests a third combination of behavioral traits showing that the aggressive behaviors often observed in autistic traits participants can in effect co-occur with ADHD-like traits. The diverse behavioral profile of each subgroup highlights the importance of combining independent behaviors into one multivariate analysis to observe how they distribute. For example, as mentioned previously, both the *Attn* and *Emot* subgroups show high levels of attention problems and hyperactivity, and remain relatively close in anxiety and depression levels. The *Emot* subgroup, however, exhibits exceptionally high levels of aggression and reactivity, a factor that separates one ADHD-like autistic traits subgroup into two (i.e. *Attn* and *Emot*), thus increasing behavioral homogeneity and the likelihood of extracting biological features from cortical images.

Notably, the unsupervised clustering analysis yielded high autistic traits subgroups with gender ratios (averaging 2:1, male to female) differing from those usually reported ASD populations (averaging 4:1, male to female)^92^. However, this gender disequilibrium is not entirely surprising as this difference can be explained by the fact that studies sampling from the general population often show a lower ratio (3:1)^93^, and that overall variability may play a role. Moreover, several studies have reported ratios ranging from 2:1 to 7:1^94–97^, indicating a heterogeneity that warrants further exploration as well as a diversity in gender ratio that depends on how cohorts are built. Most importantly, within the current investigation gender differences were controlled for in the neuroimaging analysis.

This study has several potential limitations. Firstly, though the SRS included a repetitive behavior subscale, it would have been interesting to include an independent repetitive behavior component within the clustering analysis. Concerning the unsupervised clustering, inherent limitations include the somewhat arbitrary determination of the number of clusters, and difficulties to reproduce the same partitioning in another dataset. Also, it is challenging to account for covariates in unsupervised clustering analyses. Alternative approaches could also have been applied on this dataset that would prove interesting in future studies including clustering based on SRS subscales (with subsequent study of behavioral and morphological traits), or clustering on a broader range of scales (not only pertaining to behaviors central in ASD) with subsequent isolation of subgroups high in SRS. Additionally, the present study used a general population-based cohort, and not one tailored for ASD studies, thus warranting the careful isolation of behavioral variables relevant to our objective. Within the morphometric results, it is possible we did not observe further thickness differences due to the wide age range of our cohort. Thickness changes more with age and environment and may thus present larger heterogeneities than does gyrification (which is typically developed in-utero and shortly after birth), leading us to observe greater gyrification alterations within our results^98,99^. Also, the average age of subjects in the current study (~11.4 years old) could indicate that our cortical results are consequences of differential child development, a hypothesis however that can only be confirmed using a longitudinal, prospective design. Clinical diversity in autistic traits may be further explained by other modalities, thus next steps would involve considering genetic, volumetric, diffusion, and functional differences between the acquired subgroups.

In conclusion, we showed that subtypes of autistic traits yield refined signatures and therefore stress the importance of stratification using a dimensional approach. Several studies, including the current one, have demonstrated the difficulty in yielding significant biological features in case-control comparisons, leading to large-scale inconsistencies within ASD literature. Since several of the behavioral associations and affected cortical regions discussed in this study have similarly been implicated in ASD studies, our findings maintain the growing assumption that outcomes in autistic traits are related to variations observed in ASD patients. By uncovering better-defined subtypes of ASD, studies can finally begin to truly understand the underlying genetic, biological and behavioral mechanisms of this syndrome.

## Supplementary information

Supplementary Figures

Supplementary Table
