# Supplementary material for: Cortical signatures in behaviorally clustered autistic traits subgroups: a population-based study
Source: Transl Psychiatry. 2020 Jun 27;10:207. doi: 10.1038/s41398-020-00894-3 (PMC7320967; doi:10.1038/s41398-020-00894-3)
Supplement: Supplementary file 1 — Supplementary Figures [file 41398_2020_894_MOESM1_ESM.doc]

# **Supplementary Figures**

# Optimal Cluster Selection


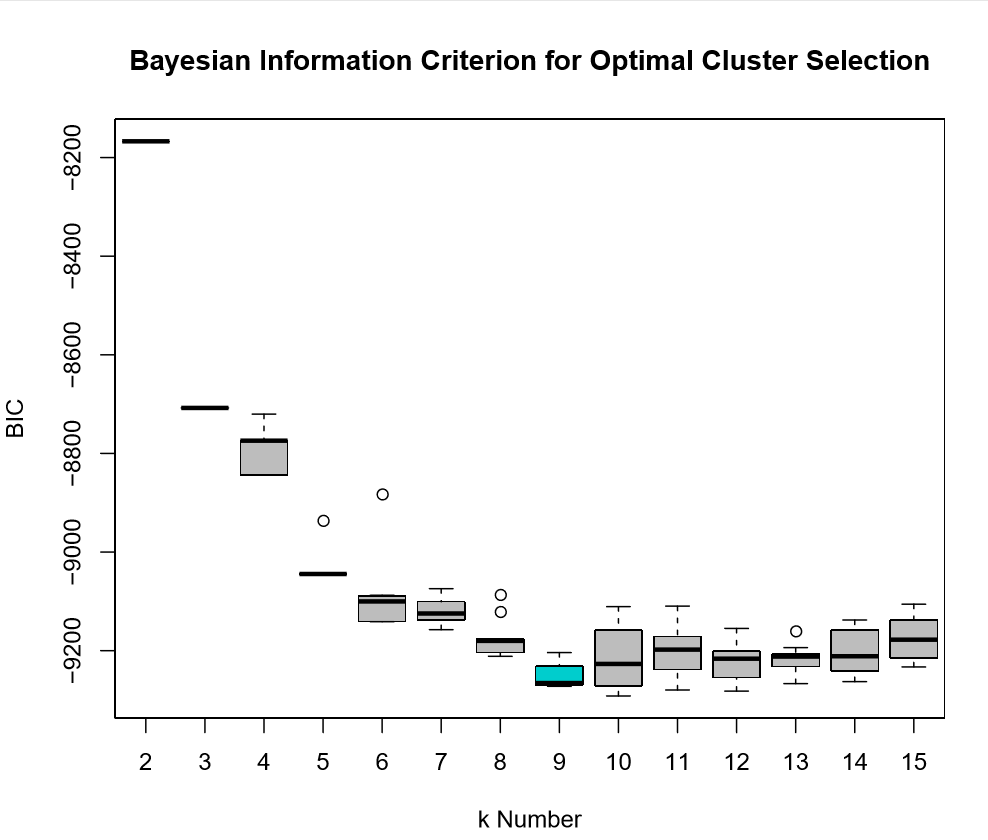

**Supplementary Figure 1: BIC Distribution of Best Cluster Options.** This curve suggests the optimal choice for k=9. We calculated Bayesian Information Criterion (BIC) distributions corresponding to clusters ranging from k=2 to k=15, in order to gain confidence in our cluster selection. Moreover this procedure was repeated with 10 different randomly chosen starting number points for each cluster number to observe and ensure validity[1].

# High Autistic Traits Subgroup Selection


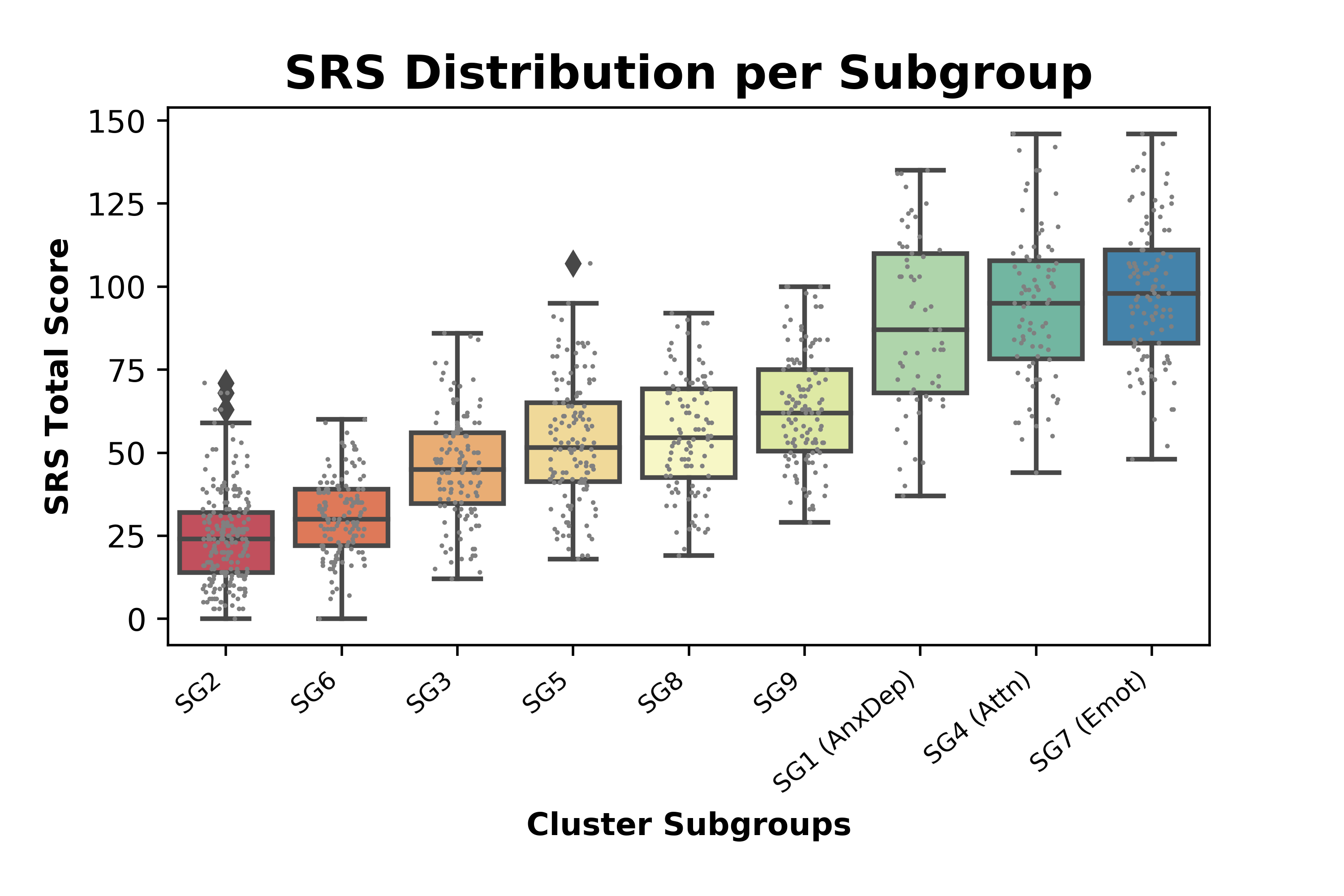

**Supplementary Figure 2: Isolation of High Autistic Traits Subgroups.** As seen in the figure, 3 subgroups (from the 9 generated in the cluster analysis) showed high levels of SRS and were thus chosen as our high autistic traits subgroups for the current study. They were chosen based on raw values falling within the ‘*severe’* or upper ‘*moderate’* classification of the SRS (above ~80), thus indicating a truly high level of social impairment and providing us with greater confidence that subjects within these subgroups are indeed representative of having high autistic traits. Each boxplot indicates the median and ranges of raw SRS scores within each subgroup. Diamonds represent outlier values.

# Principle Component Analysis (PCA) Plots of Subjects Space


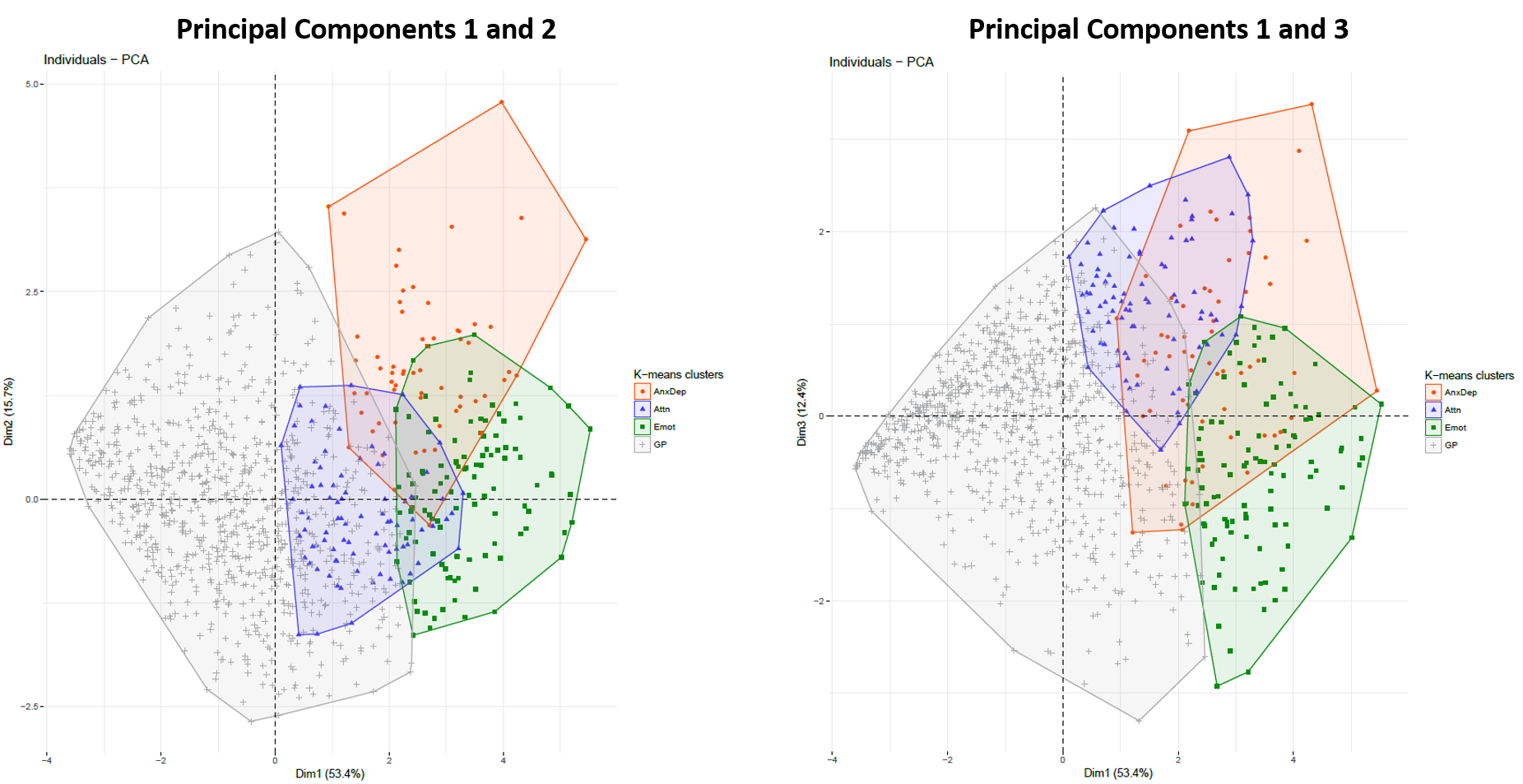
 **Supplementary Figure 3: PCA Plots of Subjects Space.** Plots representing subject variances along principle components 1 and 2, and principle components 1 and 3, showing clusters of subgroups (our high autistic subgroups and controls) based on similarity. Subjects are indicated by dots and the four clusters are represented by convex ellipses (*AnxDep = Red; Attn = Blue; Emot = Green; GP(general population/’controls’) = Grey*).

# Outlier Detection for the Morphological Analysis


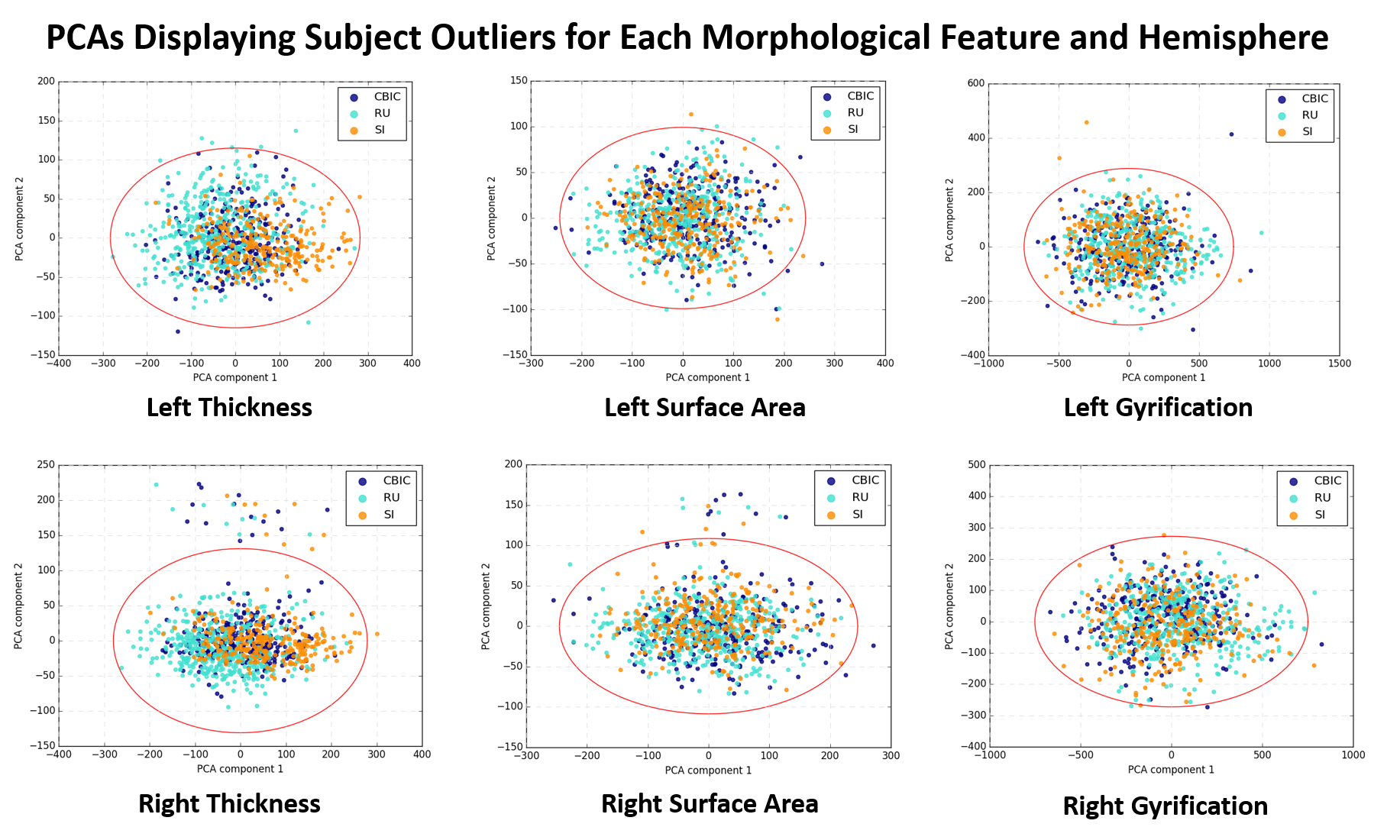
 **Supplementary Figure 4: PCA Plots Indicating Subject Outliers.** Plots illustrating outlying subjects are shown, coloured according to neuroimaging site, for each morphological feature and hemisphere. In order to perform the morphological outlier detection analysis, Hotelling’s T^2^ multivariate confidence interval was computed for each feature (i.e. thickness, surface area and gyrification) based on the first two principle components of a principal component analysis [2]. Bonferroni corrections were applied according to the presence of 3 morphological features and 2 hemispheres. Subjects were removed if they were an outlier in at least one of the morphological feature analyses (i.e. maximum 3 for each feature), causing the elimination of 23 outlying subjects. This resulted in 1 subject being removed from the *Attn* subgroup, 1 from the *AnxDep* subgroup, 2 from the *Emot* subgroup, and 19 from the controls, giving us our total of 527 subjects for the morphological analysis (in addition to the remaining subjects that were removed due to bad quality MRI scans). The three different colours represent the three neuroimaging sites (*CBIC= Cornell Brain Imaging Centre, RU= Rutgers University Brain Imaging Centre, SI= Mobile Van Staten Island*).

*REFERENCES*

1. Schwarz G. Estimating the Dimension of a Model. Ann Stat. 1978;6:461–464.

2. Hotelling H. The Generalization of Student’s Ratio. Ann Math Stat. 1931;2:360–378.
