# Supplementary material for: Cortical signatures in behaviorally clustered autistic traits subgroups: a population-based study
Source: Transl Psychiatry. 2020 Jun 27;10:207. doi: 10.1038/s41398-020-00894-3 (PMC7320967; doi:10.1038/s41398-020-00894-3)
Supplement: Supplementary file 2 — Supplementary Table [file 41398_2020_894_MOESM2_ESM.doc]

# **Supplementary Table**

# Clinical and Demographic Information on Neuroimaging Subgroups

*Values surpassing clinical levels of each score.
Ranges for each score: SRS: 0 to 123+; SCARED-p: 0 to 82; ARI-p: 0 to 12; SDQ-Hyperactivity: 0 to 10; CBCL-AB: 0 to 40+; CBCL-AP: 0 to 22+; CBCL-WD: 0 to 17+.

**Supplementary Table 1: Demographic Information for Subgroups From Neuroimaging Cohort.** Mean and standard deviation information for autistic traits subgroups on age, FSIQ, and behavioral scores, as well as gender ratio. The same information is also included for the combined *hSRS* group and the control group*. (s.d = Standard Deviation).* Clinically high values were bolded and denoted with a ‘*’ superscript.
